# Validation of predictive equations for resting energy expenditure in children and adolescents with different body mass indexes

**DOI:** 10.1186/s12937-023-00868-3

**Published:** 2023-08-10

**Authors:** Nilüfer Acar-Tek, Duygu Ağagündüz, Teslime Özge Şahin, Hatice Baygut, Elif Adanur Uzunlar, Hazal Küçükkaraca Zakkour, Ayşegül Karaçallı

**Affiliations:** 1 Faculty of Health Sciences, Department of Nutrition and Dietetics, Gazi University, Ankara, Turkey; 2 Faculty of Health Sciences, Department of Nutrition and Dietetics, Süleyman Demirel University, Isparta, Turkey; 3 Faculty of Health Sciences, Department of Nutrition and Dietetics, Karadeniz Technical University, Trabzon, Turkey; 4 Faculty of Health Sciences, Department of Nutrition and Dietetics, Ondokuz Mayıs University, Samsun, Turkey; 5 Faculty of Health Sciences, Department of Nutrition and Dietetics, Tokat Gaziosmanpaşa University, Tokat, Turkey

**Keywords:** Childhood obesity, Resting energy expenditure, Indirect calorimeter, Predictive equation

## Abstract

**Background:**

Accurate estimation of resting energy expenditure (REE) in children and adolescents is important to establish estimated energy requirements. The objective of this study was to assess the validity of existing equations in literature and a newly developed equation in estimating REE in children and adolescents.

**Methods:**

275 participants (148 boys, 127 girls) aged 6–18 years included in the study were classified as normal-weighted, overweight, obese based on BMI z-scores for age according to WHO-2007 growth curves for 5–19 years of age. REEs were measured using an indirect calorimeter, with various equations, and a newly established equation [REE = 505.412+(24.383*FFM);Adjusted R^2^ = 0.649] were compared with REE measured using Bland-Altman and further validation parameters.

**Results:**

When the predicted REEs were compared with the measured REEs, the highest prediction accuracy was achieved using the new Eq. (64.8%) and IOM (63.8%) for normal-weight participants, Müller FFM and new Eq. (59.6%) for overweight participants and Lazzer (44.9%) for obese participants. In normal and overweight participants, lowest root mean squared error (RMSE) values were acquired from Schmelzle’s equation (respectively 136.2;159.9 kcal/d), and the highest values were found in Kim’s Eq. (315.2; 295.2 kcal/d respectively). RMSE value of the new equation was 174.7 kcal/d for normal-weight children and adolescents, and 201.9 kcal/d for overweight ones. In obese participants, the lowest RMSE value was obtained from Schmelzle’s Eq. (305.4 kcal/d) and the new Eq. (317.4 kcal/d), while the highest value was obtained from IOM Eq. (439.9 kcal/d). RMSE was higher in obese groups compared to the other BMI groups.

**Conclusion:**

Indirect-calorimeter is the most suitable method for REE measurement in especially obese children and adolescents. The new equation and Schmelzle’s equation appear to be most accurate equations for normal and overweight children and adolescents.

## Background

According to the definition of the World Health Organization (WHO), energy requirement is the amount of nutrient energy required to balance energy expenditures that maintain body size, body composition, and required physical activity level for a long-term healthy life. In addition, the energy required for optimal growth and development in children should be taken into account [1]. When there is an imbalance between the nutrient requirement and intake, changes in growth and development, malnutrition, or obesity occur in children [2, 3]. Obesity is one of the most common health problems worldwide, and its prevalence has increasingly become a major public health problem globally. Children who are obese tend to be obese adults, so risk factors for weight gain are likely to exist during childhood and adolescence [4, 5]. As with adults, body-weight gain in children is the result of long-term positive energy balance that is caused by an imbalance between energy expenditure and energy intake, so that energy intake exceeds energy requirements including metabolic rate and growth [6, 7].

Correct assessment of energy requirements is required to evaluate the nutritional status of individuals and to determine the effectiveness of planned nutritional interventions [8]. The regulation of energy expenditure has been extensively researched for decades. The components of 24-hour energy expenditure or total energy expenditure (TEE) are REE, thermal effect of food, and physical activity energy expenditure. In a normal individual, approximately 60-70% of the TEE is due to REE, 10% is due to thermal effect, and 20% is due to physical activity expenditure [9]. REE is the amount of energy needed by a person to perform vital activities. With a few exceptions, REE is the largest component of total daily energy expenditure [8, 10]. Also, 60-70% of REE consists of the energy consumed by the main organs such as liver, brain, kidney, and heart [11]. In general, the basal metabolic rate (BMR) is influenced by various factors such as body composition, which is indicated by fat-free mass (FFM) and fat mass (FM) as well as age, gender, body surface, sleep, fever, environmental temperature, menstruation status, disease state, catecholamines, and some medications [12]. Body fat can independently predict the differences in resting energy expenditure (REE) among individuals, which is reasonable because adipose tissue consumes oxygen at a rate of 0.4 ml per kilogram per minute, which is significantly lower compared to the lean tissue [13]. In another study examining the factors influencing the BMR of obese and overweight children found that FFM accounted for approximately 60% of the variability in BMR. This suggests that FFM plays a significant role in determining BMR in both obese and overweight children [14].

The ‘gold standard’ method to measure REE is indirect calorimetry, in which REE is estimated from carbon dioxide and oxygen exchange measurements in vivo [15, 16]. The FitMate device developed by Cosmed which measures oxygen consumption and resting metabolic rate is frequently used in studies [17]. However, using indirect calorimetry may not be possible in all settings because it is time consuming and requires an experienced clinician and it is a costly equipment [15, 18]. Therefore, it continues to be more practical to use REE estimation equations that give the closest result with indirect calorimeter measuring devices in estimating TEE [19]. Various studies have been conducted to develop some predictive equations to predict REE, such as Harris-Benedict, Mifflin-St Jeor, WHO/Food and Agriculture Organization (FAO)/United Nations University (UNU), Muller, Owen, Schofield, and Liu formulas [15–19]. These equations are based on regression analysis of body weight, height, sex, age, lean mass, fat mass (FM), and body surface area as independent variables [20–26]. However, each equation cannot be applied to different BMI groups and different racial/ethnic groups; therefore, the characteristics of the sample population to be used should be taken into account [8, 27]. REE is affected by many factors including age, gender, body composition, ethnicity, as well as metabolic stress, muscle tone, body temperature, and drug use. Therefore, the equations used in REE calculation should be selected precisely in accordance with the characteristics of the target group [28, 29]. In clinical practice, underestimation or overestimation of energy expenditure causes insufficient dietary advice, which, together with decreased motivation of patients, can reduce dietary adherence in obese patients or lead to dietary treatment failure in malnourished patients. For this reason, it is very important to choose the best alternative equation suitable for the population of the country where the equation will be used, BMI classification, and especially for specific age groups [29].

The aim of the present study was to measure REE in children and adolescents with different BMIs by indirect calorimetry method, compare the results with REE values estimated by equations, and develop the most appropriate equation for this group.

## Methods

### Study population

This cross-sectional study was conducted between January 2019 and January 2020. Invitations were sent to the primary and secondary schools in Ankara, and 275 volunteers (148 boys, 127 girls [G*Power: 95% power at the 5% error level] aged 6–18 (11.8 ± 3.19 years) who accepted to participate were included in the study. Participants were classified as normal weight (n = 105), overweight (n = 52), and obese (n = 118) based on BMI z-scores for age according to the growth curves developed by the WHO-2007 for 5–19 years. Those with endocrine and metabolic disorders or respiratory diseases such as asthma, those with flu or colds at the time of the REE measurement, and those who regularly use medication were not included in the study.

### Ethical considerations

‘Ethics Committee Approval (2019-021)’ dated 08.01.2019 and numbered 01 was obtained from Gazi University Ethics Committee. Clear explanations were provided for the parents with regard to the purpose of the study, after which written informed consent was obtained from all the parents in accordance with the Declaration of Helsinki (World Medical Association).

### Determination of general characteristics of participants

To determine the sociodemographic characteristics and health histories of the participants, a questionnaire form was administered to the participants by face-to-face interviews.

### Body composition analysis

Anthropometric measurements and body composition analyses of the participants were taken by the researchers in accordance with their technique. The height was measured with a stadiometer with 0.5 cm sensitivity with the head in the Frankfort plane and the feet adjacent. Body composition analyses of the participants [body weight (kg), body fat mass (kg), body fat percentage (%), FFM(kg)] were performed after 8 h of fasting in the morning using bioelectrical impedance analyzer (Tanita BC-420MA (Tanita Corporation, Tokyo, Japan)). BMI value (kg/m^2^) was calculated by dividing body weight by height squared [30].

### Resting energy expenditure measurement

The REE was measured by the researchers using the indirect calorimeter method using the COSMED FitmatePro (COSMED, Rome, Italy). Measurements were taken between 08.00 and 10.00 in the morning after at least 8 h of fasting. Participants to be measured for REE were asked not to do any heavy exercise the day before. Before the test, the participants were rested for 15 min in a sitting position. The measurement was made in the supine position with a mask that completely covers the mouth and nose to determine the oxygen consumption (VO_2_) of the participants, allowing them to stand still and at rest [16].

### Equations used in estimating resting energy expenditure

In this study, a total of 13 REE calculation equations were used to compare with the REE values measured by indirect calorimetry (Table 1). Equations developed by WHO (3–10 and 10–18 years), Schofield (3–10 and 10–18 years), Institutes of Medicine (IOM) (3–18 years), Kim (4–11 years), Henry (3–10 and 10–18 years), Molnar (10–16 years), Müller (5–17 years), Derumeaux-Burel (3–18 years), Schmelzle (4–15 years), Tverskaya (6–18 years), and Lazzer (6–18 years), specifically developed for children and adolescents of different age and gender groups, were used in the study [15, 23, 25, 31–39] (Table 1).

**Table 1:** Predictive Equations for REE in Children and Adolescents Used in the Present Study

Author and Year	Gender	Age (years)	PredictiveEquationfor REE	Note
WHO, 1985	Male	3–10	kcal/d: (22.7*WT) + 495	WT:kg
	Female	3–10	kcal/d: (22.5*WT) + 499	WT:kg
	Male	10–18	kcal/d: (18.4*WT) + 651	WT:kg
	Female	10–18	kcal/d: (12.5*WT) + 746	WT:kg
Schofield, 1985	Male	3–10	kcal/d: (19.589*WT) + (1.302*HT) + 414.7	WT:kg, HT:cm
	Female	3–10	kcal/d: (16.961*WT) + (1.617*HT) + 371.0	WT:kg, HT:cm
	Male	10–18	kcal/d: (16.245*WT) + (1.371*HT) + 515.3	WT:kg, HT:cm
	Female	10–18	kcal/d: (8.361*WT) + (4.654*HT) + 200.0	WT:kg, HT:cm
IOM, 2004	Male	3–18	kcal/d: 68 - (43.3*AGE) + (7.12*HT) + (19.2*WT)	WT:kg, HT:cm, AGE:years
	Female	3–18	kcal/d: 189 - (17.6*AGE) + (6.25*HT) + (7.9*WT)	WT:kg, HT:cm, AGE:years
Kim, 2009	All	4–11	kcal/d: 632.4 + (15.66*AGE) + (9.53*WT)	WT:kg, HT:cm, AGE:years
Henry, 2005	Male	3–10	kcal/d:(15.1*WT) + (0.742*HT) + 306	WT:kg, HT:cm
	Female	3–10	kcal/d: (15.9* WT) + (2.1*HT) + 349	WT:kg, HT:cm
	Male	10–18	kcal/d:(15.6* WT ) + (2.66*HT) + 299	WT:kg, HT:cm
	Female	10–18	kcal/d:(9.40* WT) + (2.49*HT) + 462	WT:kg, HT:cm
Molnar, 1995	All	10–16	kj/d: (50.2*WT) + (29.6*HT) – (144.5*AGE) - (550*SEX) + 594.3	WT:kg, HT:cm, AGE:years, SEX: Male:0, Female:1
Müller (a), 2004	All	5–17	MJ/d: (0.02606*WT ) + (0.04129*HT)+(0.311*SEX) - (0.08369*AGE) − 0.808	WT:kg, HT:cm, AGE:years, SEX: Male:0, Female:1
Müller (b), 2004			MJ/d: (0.07885*FFM) + (0.02132*FM) + (0.327*SEX) + 2.694	FFM:kg, FM:kg, SEX: Male:0, Female:1
Derumeaux-Burel for obese children, 2004	All	3–18	MJ/d: (0.1371*FFM) - (0.1644*AGE) + 3.3647	FFM:kg, AGE:years
Schmelzle for obese children, 2004	Male	4–15	kcal/d:(6.6*WT) + (13.1*HT) – 794	WT:kg, HT:cm
	Female	4–15	kcal/d:(11.9*WT) + (0.84*HT) + 579	WT:kg, HT:cm
Tverskaya for obese children, 1998	All	6–18	kcal/d: 775+ (28.4*FFM) - (37*AGE) + (3.3*FM) + (82*SEX)	FFM:kg, FM:kg, AGE:years, SEX: Male:1, Female:0
Lazzer for obese children (a), 2006	All	6–18	kj/d:(SEX*892.68) - (AGE*115.93) + (WT*54.96) + (HT*1816.23) + 1454.50	WT:kg, HT:m, AGE:years, SEX: Male:1, Female:0
Lazzer for obese children (b), 2006	All	6–18	kj/d:(SEX*909.12) - (AGE*107.48) + (FFM*68.39) + (FM*55.19) + 3631.23	FFM:kg, FM:kg, AGE:years, SEX: Male:1, Female:0

REE: Resting energy expenditure

### Statistical analysis

All statistical analyses were performed using SPSS (Statistical Package for Social Sciences) Version 22.0 (SPSS Inc., Chicago, IL, USA). Data are presented as mean and standard deviation (SD). The normality of data distribution was evaluated using the Shapiro-Wilk or the Kolmogorov-Smirnov tests. The two-tailed Student’s t-test was used to compare differences in the mean values of normally distributed variables between male and female participants. The Mann-Whitney U test was used to compare male and female subjects for not normally distributed.

Accuracy of the predictive equations at individual and population levels were calculated. The mean percentage difference between the predicted and measured REE, respectively, was considered a measure of accuracy at group levels. The percentage of patients having a predicted REE within ±10% of the measured REE was considered a measure of accuracy at an individual level and a measured REE predicted value within 90% and 110% was considered an accurate prediction. The mean percentage difference between predicted and measured values (bias, %) and RMSE was calculated. RMSE was used to better indicate the prediction obtained with this model in our data set. Moreover, statistical analyses were performed by simple linear regression on variables related to the measured REE. Stepwise multiple regression analysis (backward selection technique) was used with the measured REE and integrating all factors for which p value in the simple linear regression was ≤0.05.

The Bland-Altman plot and analysis was used to compare the REE measured using the indirect calorimeter and calculated using different predictive equations in obese children and adolescents. Horizontal lines in the Bland-Altman plots of females and males were drawn at the mean difference and at the limits of agreement, which were defined as the mean difference plus and minus 2 times the standard deviation of the differences. A two-sided p value < 0.05 was considered significant for all analyses.

## Results

The general characteristics of the participants are presented in Table 2. Females had a significantly higher body fat (16.7 ± 9.74 kg vs. 13.9 ± 9.27 kg) as well as body fat percentage (28.5 ± 7.82% vs. 22.2 ± 9.59%) and significantly lower FFM (37.1 ± 10.58 kg vs. 45.6 ± 15.37 kg) (*p* < 0.05, Table 2).

**Table 2:** The main characteristics of subjects

	Total (n = 275)	Male (n = 148)	Female (n = 127)	
Variables	**Mean ± SD**	**Mean ± SD**	**Mean ± SD**	**p value**
Age (year)	11.8 ± 3.19	12.1 ± 3.35	11.5 ± 2.97	NS
Body weight (kg)	57.1 ± 20.71	59.8 ± 21.26	53.9 ± 19.67	p < 0.01*
Height (cm)	153.0 ± 17.11	156.1 ± 18.93	149.4 ± 13.95	p < 0.001*
BMI (kg/m^2^)	23.6 ± 5.81	23.8 ± 5.58	23.4 ± 6.09	NS
BMI (z score)	1.6 ± 1.57	1.6 ± 1.52	1.6 ± 1.63	NS
Fat percentage (%)	25.1 ± 9.34	22.2 ± 9.59	28.5 ± 7.82	p < 0.001*
FM (kg)	15.2 ± 9.58	13.9 ± 9.27	16.7 ± 9.74	p < 0.01*
FFM (kg)	41.6 ± 14.01	45.6 ± 15.37	37.1 ± 10.58	p < 0.001*
Measured REE(kcal/day)	1521.8 ± 423.69	1625.8 ± 486.00	1400.5 ± 295.46	p < 0.001*
VO_2_ (ml/min)	218.5 ± 60.92	233.5 ± 69.80	200.9 ± 42.57	p < 0.001*
Rf (l/min)	18.7 ± 4.39	18.7 ± 4.61	18.6 ± 4.12	NS
FeO_2_ (%)	17.0 ± 0.62	17.0 ± 0.61	17.0 ± 0.64	NS

BMI = body mass index, FM = fat mass, FFM = fat-free mass, REE = resting energy expenditure, VO_2_ = The average oxygen consumption (in ml per minute), Rf = The average respiratory frequency, FeO_2_ = The average oxygen concentration in the exhaled air

NS = Not significant

*The difference between male and female groups were found statistically significant (p < 0.05)

There was a statistically significant difference in measured REE (M: 1625.8 ± 486.00 kcal/d vs. F: 1400.5 ± 295.46 kcal/d) and VO2 (M: 233.5 ± 69.80 mL/min vs. F: 200.9 ± 42.57 mL/min) between the two genders (*p* < 0.05), but not Rf (L/min) or FeO2 (%) (*p* > 0.05, Table 2).

Table 3 presents the difference between predicted and measured REE (kcal/d), prediction accuracy (%), bias (%), and RMSE (kcal/d) values for all participants. For all participants, Schmelzle’s equation had the lowest and IOM equation had the highest RMSE value (227.9 kcal/d and 327.2 kcal/d, respectively; Table 3). Prediction accuracy varied between 33.3% (Molnar’s equation) and 63.8% (IOM equation) in normal-weight individuals, between 38.5% (Kim’s equation) and 59.6% (Müller FFM) in overweight individuals, and between 28.0% (Kim’s equation) and 44.9% (Lazzer equation) in obese individuals. Moreover, the lowest and highest RMSE values were obtained with Schmelzle’s (136.2 kcal/d) and Kim’s equations (315.2 kcal/d) in normal-weight individuals, Schmelzle’s (159.9 kcal/d) and Kim’s (295.2 kcal/d) equations in overweight individuals, and Schmelzle’s (305.4 kcal/d) and IOM (439.9 kcal/d) equations in obese individuals (Table 3).

**Table 3:** Evaluation of REE with different predictive equations in overall children and adolescents with different BMIs based on differences predicted-measured, percentage of accuracy, bias, and RMSE

	Total (n = 275)				Normal Weight (n = 105)				Overweight (n = 52)				Obese (n = 118)			
REE predictive equations	Difference predicted-measured (kcal/d)	Accurate-prediction (%)	Bias (%)	RMSE (kcal/d)	Difference predicted-measured (kcal/d)	Accurate-prediction (%)	Bias (%)	RMSE (kcal/d)	Difference predicted-measured (kcal/d)	Accurate-prediction (%)	Bias (%)	RMSE (kcal/d)	Difference predicted-measured (kcal/d)	Accurate-prediction (%)	Bias (%)	RMSE (kcal/d)
Tverskaya	93	50.9	9.9	282.4	6	61.9	3.1	179.9	69	53.8	6.2	210.5	182	39.8	17.5	370.8
Derumeaux-Burel	182	43.6	15.8	320.5	103	55.2	9.7	196.4	169	40.4	12.1	264.0	259	34.7	22.6	417.6
Müller	-49	44.0	1.33	297.3	-57	46.7	0.1	255.3	-69	55.8	-1.2	251.6	-33	36.4	3.5	346.6
Müller FFM	27	49.8	5.8	268.2	-7	53.3	2.9	215.5	17	59.6	3.4	208.8	62	42.4	9.4	327.2
Schmelze	-10	50.9	2.7	227.9	-39	59.0	-0.8	136.2	-51	57.7	-1.6	159.9	15	40.7	5.7	305.4
Molnar	-50	37.5	0.9	294.3	-159	33.3	-7.6	281.4	-84	48.1	-2.0	246.0	40	36.4	8.0	323.6
H	-47	48.7	-0.6	267.6	-105	58.1	-5.6	219.2	-68	46.2	-3.2	214.9	13	41.5	5.0	322.2
IOM	65	45.8	7.8	327.2	-36	63.8	-0.1	184.0	53	44.2	4.9	242.5	160	30.5	16.1	439.9
Schofield	29	50.5	5.3	272.6	-54	59.0	-1.2	198.4	-4	55.8	1.4	197.0	119	40.7	13.0	348.0
WHO/FAO/UNU	67	49.1	8.1	287.1	-52	56.2	-0.7	204.2	24	57.7	3.6	194.9	193	39.0	18.0	371.8
Kim	-104	36.0	-3.3	328.4	-64	43.8	-2.7	315.2	-163	38.5	-8.8	295.2	-106	28.0	-2.2	352.8
Lazzer	23	47.3	5.6	289.2	-78	48.6	-1.9	238.6	-24	50.0	0.9	213.2	134	44.9	14.4	352.5
Lazzer FFM	41	42.9	7.2	298.8	-68	45.7	-0.5	254.4	-9	46.2	2.1	218.2	161	39.0	16.4	359.8
New Equation	0	52.7	3.0	250.1	-12.9	64.8	0.9	174.7	3.69	59.6	1.48	201.9	9.85	39.0	5.7	317.4

Table 4 presents the difference between predicted and measured REE (kcal/d), prediction accuracy (%), bias (%), and RMSE (kcal/d) values for different genders and BMI groups. Prediction accuracy ranged between 24.1% (Kim’s equation) and 63.0% (IOM equation) for normal-weight females and between 37.3% (Molnar’s equation) and 64.7% (IOM, Schofield’s and Kim’s equations) for normal-weight males, between 25.9% (Derumeaux-Burel’s equation) and 66.7% (Schmelzle’s equation) for overweight males and between 44.0% (Molnar’s and Kim’s equations) and 60.0% (Tverskaya and Müller equations) for overweight females, and between 25.4% (IOM and Kim’s equations) and 41.8% (Lazzer and Schofield’s equations) for obese males and between 31.4% (Kim’s equation) and 58.8% (Derumeaux-Burel’s equation) for obese females (Table 4). The comparison of RMSE values of different equations and groups revealed that RMSE was often higher for males than for females. RMSE was higher in obese individuals compared to the other BMI groups. Moreover, for obese males and females, RMSE was lowest in Müller FFM (367.7 kcal/d) and Schmelzle’s (262.7 kcal/d) equations, respectively, and highest in Derumeaux-Burel’s Eq. (446.3 kcal/d and 376.7 kcal/d, respectively).

**Table 4:** Evaluation of REE with different predictive equations in overall children and adolescents with different BMIs based on differences predicted-measured, percentage of accuracy, bias, and RMSE according to genders (M/F)

	Normal Weight (n = 105)				Overweight (n = 52)				Obese (n = 118)			
REE predictive equations	Difference predicted-measured (kcal/d)	Accurate-prediction (%)	Bias (%)	RMSE (kcal/d)	Difference predicted-measured (kcal/d)	Accurate-prediction (%)	Bias (%)	RMSE (kcal/d)	Difference predicted-measured (kcal/d)	Accurate-prediction (%)	Bias (%)	RMSE (kcal/d)
Tverskaya	21/-10	61.1/62.7	5.0/1.1	202.5/152.4	120/13	48.1/60.0	9.2/2.9	231.8/184.7	209/147	38.8/41.2	21.0/12.9	412.7/307.2
Derumeaux-Burel	108/98	55.6/54.9	9.7/9.7	210.7/179.9	212/124	25.9/56.0	14.2/10.6	295.9/224.4	261/255	29.9/58.8	24.4/20.4	446.3/376.7
Müller	-181/74	35.2/58.8	-7.2/7.9	316.9/166.8	-172/42	51.9/60.0	-7.4/5.3	296.8/191.0	-56/-3	29.9/45.1	4.2/2.6	397.3/265.7
Müller FFM	-102/92	51.9/54.9	-2.9/9.0	246.7/176.6	-40/79	63.0/56.0	-0.4/7.6	200.2/217.7	56/71	40.3/45.1	10.8/7.6	367.7/264.7
Schmelze	40/-95	55.6/62.7	6.0/-5.6	196.2/173.7	29/-123	66.7/48.3	3.6/-6.3	187.0/214.1	33/-8	38.8/43.1	8.5/2.1	341.5/262.7
Molnar	-259/-40	29.6/37.3	-12.1/-2.2	332.2/214.8	-172/11	51.9/44.0	-7.1/3.5	271.0/215.7	61/13	37.3/41.2	11.5/3.5	356.4/274.6
H	-150/-57	42.6/74.5	-8.3/-2.7	262.0/162.1	-66/-69	44.4/48.0	-3.8/-2.6	224.4/204.2	29/-7	41.8/41.2	6.8/2.5	358.5/267.0
IOM	-22/-51	63.0/64.7	1.7/-2.1	203.3/161.0	125/-23	40.7/48.0	9.4/0.1	238.6/246.7	293/-14	25.4/37.3	26.9/1.9	528.4/284.6
Schofield	-62/-46	53.7/64.7	-0.4/-2.0	230.3/157.6	45/-57	59.3/52.0	4.6/-1.8	194.0/200.1	215/-6	41.8/39.2	20.9/2.6	397.3/269.8
WHO/FAO/UNU	-82/-20	53.7/58.8	1.7/0.3	240.4/157.1	43/5	59.3/56.0	4.5/-1.8	195.9/193.9	252/116	38.8/39.2	23.3/11.1	416.0/304.1
Kim	-42/-80	24.1/64.7	-0.6/-4.2	408.0/166.4	-225/-105	33.3/44.0	-12.9/-5.0	345.9/228.0	-147/-52	25.4/31.4	-3.2/-1.0	404.7/269.9
Lazzer	-72/-85	42.6/54.9	0.9/-5.0	284.9/176.8	1/-52	48.1/52.0	3.3/-1.6	231.8/191.1	188/64	41.8/49.0	19.9/7.1	400.1/278.0
Lazzer FFM	-65/-72	40.7/51.0	2.2/-3.5	310.2/177.0	22/-44	44.4/48.0	4.9/-0.9	240.7/191.0	214/92	35.8/43.1	21.9/9.1	412.8/275.3
New Equation	-29/4	61.1/68.6	0.3/1.5	191.4/155.0	19/-13	77.8/40.0	2/0.8	193.4/210.7	-3/26	38.8/39.2	6.5/4.5	349.3/269.6

Figure 1 shows Bland-Altman plots indicating the differences and lower and upper limits of agreement for predicted (with different equations) and measured REE (with indirect calorimetry) among individuals with different BMIs. The Bland-Altman plots indicated that the differences did not show random distribution, and thus were not suitable for application. On the other hand, the equations showed a relatively random distribution in overweight individuals whereas they were particularly unsuitable for REE estimation in obese populations (Fig. 1).

**Fig. 1:**
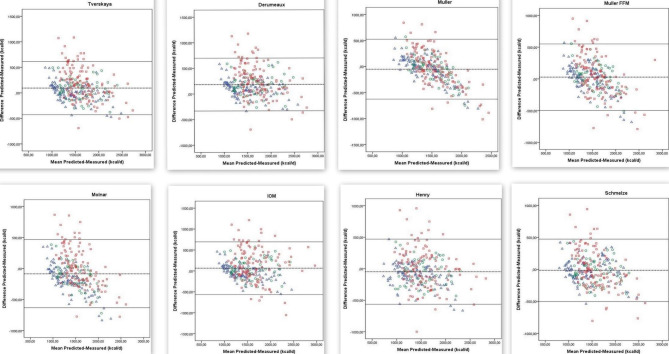
Bland-Altman plot of differences in REE measured using the indirect calorimeter and calculated using 14 different predictive equations in children and adolescents with different BMIs. Dotted lines represent 2 SDs from the mean (limits of agreement)

**Fig. 1:**
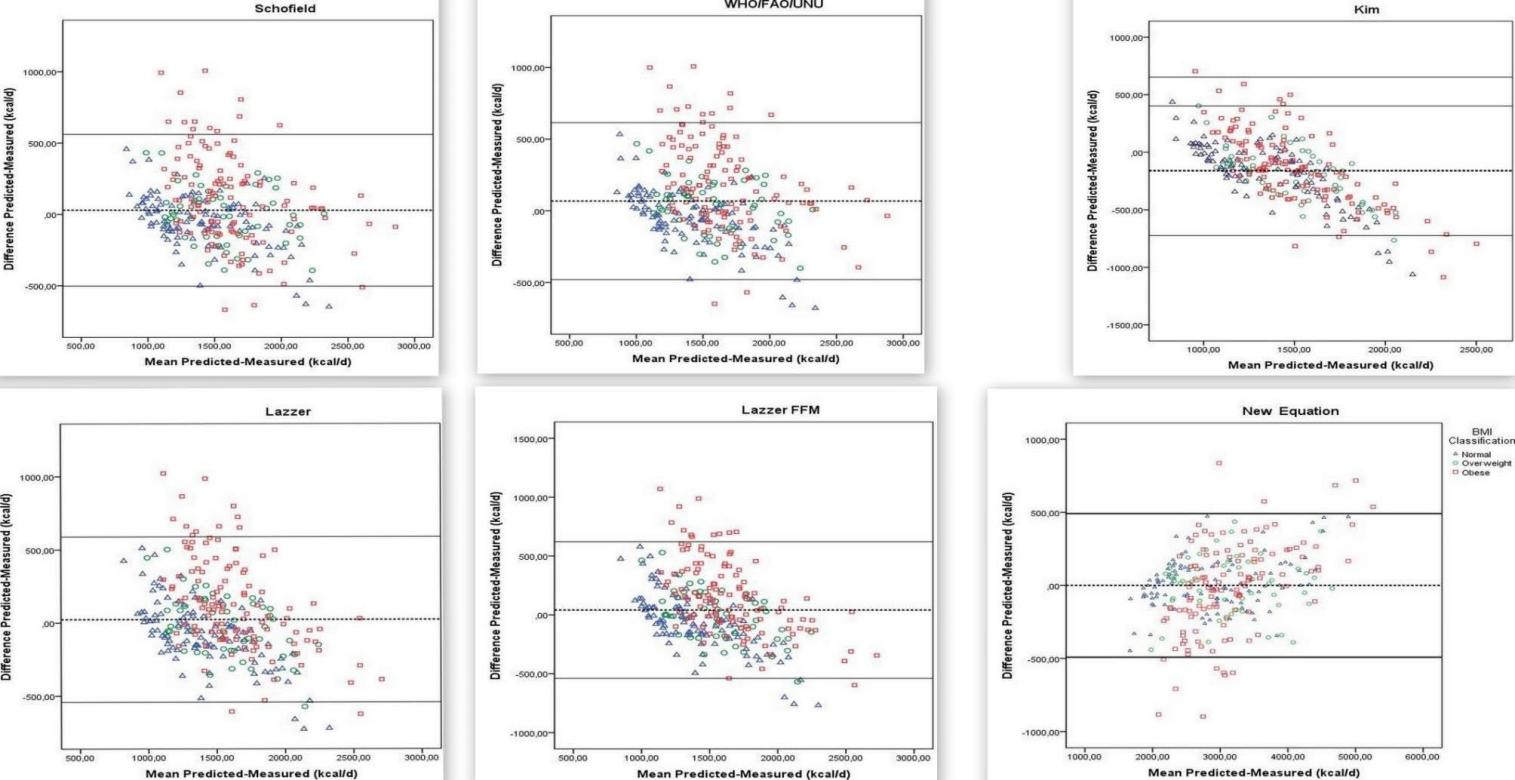
(continued) Bland-Altman plot of differences in REE measured using the indirect calorimeter and calculated using 14 different predictive equations in children and adolescents with different BMIs. Dotted lines represent 2 SDs from the mean (limits of agreement)

### Development and validation of the new REE prediction equation

Firstly, REE was accepted as the dependent variable, and all variables presented in Table 2 as independent variables. A univariate regression analysis was performed for each variable. All variables except body fat percentage (%) were significant (*p* < 0.05, Table 5). The significant variables were included in the multiple regression analysis model. In multivariate analysis with backward selection, FFM was the only variable left in the model. The results of multiple regression analysis are presented in Table 6. The equation and results taken from Table 6 are given below.

**Table 5:** Results of univariate regression analysis

Variable	Coefficients (95% CI)	Adjusted R^2^	p value
Gender	-0.225.212 (-322.664; 127.760)	0.067	p ≤ 0.001
Age (years)	81.447 (68.972; 93.921)	0.375	p ≤ 0.001
Body weight (kg)	15.171 (13.537; 16.805)	0.549	p ≤ 0.001
Height (cm	18.522 (16.566; 20.478)	0.558	p ≤ 0.001
BMI (kg/m^2^)	32.971(25.230; 40.711)	0.202	p ≤ 0.001
FFM (kg)	24.383 (22.251;26.515)	0.649	p ≤ 0.001
FM (kg)	19.109 (14.360; 23.857)	0.184	p ≤ 0.001
Body fat percentage (%)	3.033 (-2.355;8.422)	0.001	0.269

CI = confidence interval, BMI = body mass index, FFM = fat-free mass

**Table 6:** Result of multiple regression analysis^*^

Variable	Coefficients (95% CI)	p value
Constant	505.412 (411.689; 599.136)	p ≤ 0.001
FFM	24.383 (22.251; 26.515)	p ≤ 0.001

CI = confidence interval, FFM = fat-free mass

^*^ Adjusted R^2^ for the model = 0.649

$$New{\text{ }}Prediction{\text{ }}Equation:REE = {\text{ }}505.412{\text{ }} + {\text{ }}24.383{\text{ }}x{\text{ }}FFM{\text{ }}{\left( {kg} \right)^{}}$$

The intra-group/ internal cross-validation results of the newly developed equation are presented in Table 3 [37]. Accordingly, for the overall child and adolescent population, the difference between predicted and measured REE was 0 kcal/d, prediction accuracy 52.7%, bias 3.0%, and RMSE 250.12 kcal/d (Table 3). The comparison of the difference between predicted and measured REE (kcal/d), prediction accuracy (%), bias (%), and RMSE values (kcal/d) according to BMI groups revealed that the difference between predicted and measured REE was the lowest in the overweight group (3.69 kcal/d), and bias and RMSE were the lowest in the normal-weight group (0.9% and 174.7 kcal/d, respectively).

The comparison of the difference between predicted and measured REE (kcal/d), prediction accuracy (%), bias (%), and RMSE values (kcal/d) in different genders and BMI groups revealed that for both genders, the obese population had the lowest prediction accuracy (M: 38.8%, F: 39.2%) and the highest bias (M: 6.5%, F: 4.5%) and RMSE (M: 349.3, F: 269.6). For female subjects, the lowest RMSE value (155.0 kcal/d) and the highest prediction accuracy (68.6%) were in the normal-weight group, and the lowest bias (0.8%) was in the overweight group. For male subjects, the highest prediction accuracy (77.8%) was in the overweight group, and the lowest bias (0.3%) and RMSE values (191.4 kcal/d) were in the normal-weight group (Table 4).

## Discussion

In this study, we investigated the accuracy of existing REE estimation equations in children and adolescents (6–18 years) with different BMIs and found that the predicted and actual REE values were significantly different and that these equations usually had a high bias. The difference between predicted and actual REE values and the bias varied according to sex and BMI. To the best of our knowledge, this is the first study of such kind that included Turkish children and adolescents of all BMIs, not only the obese.

Nutritional problems and obesity are common public health problems that affect children and adolescents [36]. Accurate measurement of REE is essential for nutritional assessment and determining TEE [40]. TEE is composed of REE, TEF (also referred to as diet-induced thermogenesis, DIT), and activity energy expenditure [41]. Energy expenditure and its specific components can be measured using activity monitors, including direct and indirect calorimetry, isotope dilution (mainly the doubly-labeled water technique), 24 h heart rate measurements, and accelerometry, and others [42]. Indirect calorimetry is the gold standard for estimating REE [40]. Indirect calorimetry measures energy expenditure, including REE, DIT, and physical activity, based on heat production calculated from the rates of respiratory gas exchange. However, this method is disadvantageous due to its high cost and impractical nature. Therefore, since the 20th century, researchers have developed equations to estimate REE based on age, sex, race, body weight, height, and BMI [38]. Predictive equations are used in estimating REE as well as TEE based on the measurements of a large population [42]. In clinical practice, REE prediction mostly uses equations described by Harris-Benedict, Schofield, Owen, and WHO/FAO/UNU. The Mifflin-St Jeor formula is reported to be the most reliable equation for non-obese and obese adults (with a low error within 10% in estimation), but further and more detailed research is needed for its applications in different age and ethnic groups [8]. In addition, even though many equations are valid for adults, there are limited data on their validity in predicting energy expenditure (REE and TEE) in children and adolescents [39, 42]. The new prediction equations enable physicians to accurately and sufficiently estimate REE in obese children and adolescents. For validation study, internal validation, not crossover validation, was conducted. The sample size of the validation study of the new predictive equation was comparable to that of the majority of earlier studies, despite the fact that the study group was not entirely representative of all obese children and adolescents in Turkey [39]. Harris–Benedict and the Mifflin equations were the most accurate one in predicting RMR among obese and non-obese adults in validation studies [18, 43].

In this study, we evaluated the validity of several REE estimation equations and had two major findings: (1) all REE prediction equations had low prediction accuracy and high bias and RMSE, and (2) the differences were non-randomly distributed in the Bland-Altman analysis (Tables 3 and 4; Fig. 1). In this study, prediction accuracy was 50.9% at the highest for the entire study population (Table 3). Moreover, prediction accuracy ranged between 33.3% and 63.8% for normal-weight and 38.5% and 59.6% for overweight children and adolescents and was comparatively low for obese subjects (28.0-44.9%) (Table 3). One study stated that indirect calorimetry is the best option for REE measurement in overweight and obese adolescents and that Molnar equation had the highest accuracy in REE estimation in overweight and obese adolescents aged 12–18 years [44]. A different study compared predicted and measured REEs among severely obese Italian adolescents aged 14–18 years and reported that Lazzer (for both genders) and Schmelzle’s and Henry (only for females) equations could be used to estimate REE with a predicted-measured difference within ± 10%. However, considering all predictive equations, accuracy did not reach 50% in the individual patient. The study concluded that the studied equations inaccurately estimated REE particularly in obese adolescents with BMI ≥ 45.0 kg/m^2^ [45]. A study on 226 severely obese Canadian adolescents (15.9 ± 1.9 years) found that the Mifflin-St Jeor equation was the most accurate, and the REE predicted using this equation was within ± 10% of the measured REE in 61% of the participants [40]. In the present study, the equation with the lowest prediction accuracy, especially in overweight and obese groups, was Kim’s Eq. (2012) (Table 3). Kim’s equation was developed in 2012 to determine the energy requirement of Korean children and adolescents (7–18 years) [15]. We ascribe the low accuracy of Kim’s equation in our study to the differences between study populations. Consistently, a study on 502 black and white children demonstrated that race must be taken into account to accurately estimate REE using gender-specific equations in children [46].

In this study, the Henry and Molnar equations had the lowest (-0.6% and 0.9%, respectively), and the Derumeaux-Burel’s equation had the highest bias (15.8%) (Table 3). As a matter of fact, Derumeaux-Burel’s equation had the highest bias in all BMI groups and both genders (Table 3). Derumeaux-Burel’s equation was developed in 2004 for obese children aged 3–18 years [35]. Derumeaux-Burel reported that this equation was sufficient and acceptable for predicting REE in the obese pediatric population by clinicians, but our results did not confirm this proposition, even in obese subjects. In their validation study, Derumeaux-Burel et al. also indicated that published equations except for the WHO equation contained systematic bias, but our findings also failed to confirm the proposition that the WHO equation does not contain bias [35]. In our study, Schmelzle’s equation had the lowest and IOM equation the highest RMSE value for all participants (227.9 kcal/d and 327.2 kcal/d, respectively; Table 3). Furthermore, Schmelzle’s equation had the lowest RMSE for all BMI subgroups. Consistently with its low accuracy, Kim’s equation had the highest RMSE values for the normal-weight and overweight groups, and the IOM equation had the highest RMSE in the obese population (Table 3). A previous study by our research group similarly demonstrated that Schmelzle’s equation had the lowest RMSE value (331 kcal/d) for Turkish obese children and adolescents (7–17 years) [39]. Schmelzle’s equation was developed in 2004 in 82 healthy obese German children (49 males and 33 females, aged 4–15 years) [36]. The racial proximity between European and Turkish populations may explain the high accuracy of Schmelzle’s equation and the low accuracy and high RMSE value of IOM in our study.

In this study, RMSE values were higher, i.e., accuracy was lower, in male subjects for all equations (Table 4). The literature indicates that sex differences in metabolic physiology and body composition, including body fat mass and FFM, result in differences in REE [47]. Similar to our results, studies report that even the most accurate equations yield different results for the two genders [48].

In this study, we also investigated the validity of equations developed specifically for the obese pediatric population, such as the equations described by Tverksya (1998), Schmelzle (2004), Derumeaux-Burel (2004), and Lazzer (2006), and found that their prediction accuracies were low and bias and RMSE values were high compared to the results of normal- and overweight individuals (Tables 3 and 4). Outliers in the Bland-Altman analysis were determined only in obese children and adolescents. As we stated in our study, the new equation seems to be one of the most accurate equations for normal and overweight children and adolescents (Fig. 1). Most equation validation studies focus on obese children and adolescents [15, 38, 45]. One study of 264 obese Italian adolescents (14–18 years) found that the Lazzer equation was the most accurate in males, and the Henry-1, WHO/FAO/UNU, Schmelzle, and Lazzer equations in females [45]. Another study of 52 obese Korean children reported that the Molnar, Mifflin-St Jeor, Liu, and Harris-Benedict equations were the most accurate equations [15]. We ascribe our finding of low accuracy in obese subjects for the equations that were specifically developed for the obese pediatric population to genetic and racial differences.

Since none of the prediction equations were appropriate for the pediatric population, we established a new prediction equation for children and adolescents with different BMIs. In this new equation, FFM was the major predictor of REE, as expected, and explained 65% of REE [39]. Similar to our study, Müller et al. (2004) found that FFM is one of the most important determinants of REE, and that FFM alone explains 61.7% of the variance in REE in adults [23]. FFM better explains REE than body weight, and body composition is not considered to be particularly important in the pediatric population [38, 49]. However, differences in body composition (e.g., FFM and FM) are the reason behind individual differences in REE [50]. Lean body mass (LBM) and/or FFM are more metabolically active than adipose tissue, and are determinants of energy requirement [51]. For this reason, they often correlate better with REE compared to classical anthropometric measurements such as body weight and height. This may result in a bias in overweight and obese individuals [52]. One study suggested that including DXA-derived LBM to REE estimation in boys (11–15 years old) and girls (4–10 years old) could help prevent systematic error [37]. One study emphasized using compartment-specific lean mass [high-metabolic rate‐at‐rest trunk lean mass (TrLM) and low‐metabolic‐rate‐at‐rest appendicular lean mass (AppLM)] when determining energy expenditure in children of different races [51]. However, even the newly developed equation was less accurate in the obese subgroup compared to the other BMI groups. This sheds light on the need to evaluate additional parameters (such as advanced segmental analysis) in the obese population.

## Conclusion

As a result, for the estimation of REE is extremely important to determine the most appropriate predictive equation when the direct calorimeter cannot be reached. However, it is indicated that the REE estimation equations in the clinic are also limited in explaining all individual factors, especially in obese children and adolescents. This study showed that there is a wide variation in the accuracy of predictive equations.

The use of equations in estimating REE in clinical practice is a cheap and fast method, and there is a need for the use of accurate equations. However, inaccurate REE estimation in clinical intervention may lead to malpractices in medical diet therapy in obese children. For this reason measurement of REE by indirect calorimetry should be preferred, especially in obese children, instead of equations. However, in cases where an indirect calorimeter cannot be reached, it may be recommended to use the new equation and Schemelze’s equation, since it makes a closer estimate than indirect calorimetry.

## Data Availability

Not applicable.

## Abbreviations

FFM: Fat Free Mass
FM: Fat Mass
IOM: The Institute of Medicine
REE: Resting Energy Expenditure
RMSE: Root Mean Square Error
TEE: Total Energy Expenditure
TEF: The Thermic Effect of Food
